# Spatial distributions of *Anopheles* species in relation to malaria incidence at 70 localities in the highly endemic Northwest and South Pacific coast regions of Colombia

**DOI:** 10.1186/s12936-016-1421-4

**Published:** 2016-08-11

**Authors:** Martha L. Ahumada, Lorena I. Orjuela, Paula X. Pareja, Marcela Conde, Diana M. Cabarcas, Eliana F. G. Cubillos, Jorge A. Lopez, John C. Beier, Sócrates Herrera, Martha L. Quiñones

**Affiliations:** 1 Grupo de Entomología, Instituto Nacional de Salud, Bogotá, DC Colombia; 2 Departamento de Salud Pública, Facultad de Medicina, Universidad Nacional de Colombia, Bogotá, DC Colombia; 3 Secretaria de Desarrollo de la Salud de Córdoba, Córdoba, Colombia; 4 Caucaseco Scientific Research Center/Immunology Institute, Universidad del Valle, Cali, Colombia; 5 Instituto Departamental de Salud de Nariño, Nariño, Colombia; 6 Department of Public Health Sciences, Miller School of Medicine, University of Miami, Miami, FL USA

**Keywords:** *Anopheles*, Cytochrome *c* oxidase I, Colombia, Malaria

## Abstract

**Background:**

A proper identification of malaria vectors is essential for any attempt to control this disease. Between 40 and 47 *Anopheles* species have been recorded in Colombia, and eight species complexes have been identified in the last decade. An update of *Anopheles* species distribution and its relationship with malaria is required, particularly for newly identified members of species complexes.

**Methods:**

A cross-sectional entomological study was conducted at 70 localities in the highest malaria transmission areas in Colombia. In each locality, immature and adult mosquitoes were collected. All specimens were determined using morphological characters and confirmed used restriction profiles of Internal Transcribed Spacer 2 (PCR–RFLP-ITS2), and Cytochrome *c* Oxidase I (COI) sequence gene. To detect natural *Plasmodium* infections, enzyme-linked immunosorbent assay and nested PCR analysis were used. Distribution of *Anopheles* species was spatially associated with malaria incidence.

**Results:**

A total of 1736 larvae and 12,052 adult mosquitoes were determined in the 70 localities. Thirteen *Anopheles* species were identified. COI sequence analysis suggested 4 new lineages for Colombia: for *Anopheles albimanus* (*An. albimanus* B), *Anopheles pseudopunctipennis* s.l., *Anopheles neivai* (*An. neivai nr*. *neivai* 4), and *Anopheles apicimacula*. Two members of species complexes were identified, as: *Anopheles nuneztovari* C, and *Anopheles albitarsis* I. Another seven species were confirmed. Four mosquitoes were infected with *Plasmodium* species, *An. albimanus* B and *An. nuneztovari* C. In Northwest of Colombia, *An. nuneztovari* C, *An. albimanus,* and *Anopheles darlingi* were present in the municipalities with highest annual parasitic index (API) (>35 cases/1000 inhabitants). In the north of South Pacific coast, with a similar API, *An. nuneztovari* C were widely distributed inland, and the main species in coastal regions were *An. albimanus* B and *An. neivai* s.l. In the South Pacific coast bordering with Ecuador, 3 *Anopheles* species were found in municipalities with high API (15–88 cases/1000 inhabitants): *An. albimanus* B, *Anopheles calderoni* and *An. neivai* s.l.

**Conclusions:**

In the highest malaria areas of Colombia, 13 *Anopheles* species and four new lineages were found, which highlights the need for updating the species distribution. A DNA barcode analysis allowed the taxonomic identification to be refined, particularly for species complexes, and to improve the further understanding of their relation with malaria transmission.

**Electronic supplementary material:**

The online version of this article (doi:10.1186/s12936-016-1421-4) contains supplementary material, which is available to authorized users.

## Background

According to the 2015 World Malaria Report [1], 17 % of malaria cases reported in the Americas region were from Colombia, where malaria transmission exhibits an endemic/epidemic pattern that maintains unstable endemic transmission levels throughout the country [2]. A decreasing trend in clinical malaria cases has been reported over the past 14 years in Colombia, falling from 144,432 in 2000 [2] to 40,768 in 2014, with a more than 75 % decrease in the incidence of microscopically confirmed malaria during this period of time [1]. Despite this, malaria remains one of the foremost public health concerns in the western region of Colombia where more than 85 % of malaria cases were reported from 2010 to 2015 [3].

The last review of the geographical distribution of *Anopheles* species in Colombia was published over 4 years ago and mentioned the presence of 40–47 *Anopheles* species including those belonging to species complexes [4]. Many of the records included in this review took into account data collected more than 50 years ago by the Malaria Eradication Service (Servicio de Erradicacion de la Malaria, SEM).

Environmental, social, economic, and demographic changes have occurred since that time which may have substantially influenced the observed distribution of *Anopheles* species in Colombia. In addition, it is necessary to differentiate the members of the *Anopheles* species complexes, which can now be achieved using several molecular tools [5]. A clear example is the recent analysis, based on the sequencing of the mitochondrial DNA marker, Cytochrome *c* Oxidase I (COI); which showed the presence of two species of the *Anopheles (Nyssorhynchus) albitarsis* complex in Colombia: *An. (Nys.) albitarsis* F, in the departments of Meta, Norte de Santander and Vichada; and *An. (Nys.) albitarsis* I, in departments of Antioquia and Norte de Santander [6]. Based on sequences of molecular markers, COI, and internal transcribed spacer 2 (ITS 2), analysis showed that there are three species of the complex *oswaldoi*-*konderi* in Colombia: *Anopheles (Nys.) oswaldoi* A in department of Amazonas; *An. (Nys.) oswaldoi* B in departments of Antioquia, Caqueta, Meta, Norte de Santander and Putumayo; and *Anopheles sp. nr. konderi* in department of Caqueta [7]. However, there are still gaps in knowledge about the distribution of *Anopheles* mosquitoes in Colombia, as well as the presence of *Anopheles* species of other species complexes [8].

Of *Anopheles* species reported in Colombia, 12 have been implicated in malaria transmission. The main malaria vectors are *Anopheles (Nys.) darlingi*, *Anopheles (Nys.) nuneztovari* s.l., and *Anopheles (Nys.) albimanus* [9]. Other species are referred to as vectors of local importance in some areas, or are suspected of being associated with malaria transmission, such as: *Anopheles (An.) pseudopunctipennis* s.l.; *Anopheles (An.) punctimacula* s.l.; *Anopheles (An.) calderoni*; *Anopheles (An.) neomaculipalpus*; *Anopheles (Kerteszia) pholidotus*; *Anopheles (Ker.) neivai* s.l.; *Anopheles (Nys.) rangeli*; *Anopheles (Nys.) benarrochi* B; and *An.* (*Nys.*) *oswaldoi* s.l. [9–14].

Studies of malaria vector species in Colombia have usually been performed in just a few localities and usually with the aim to clarify taxonomic identifications or to understand the biology, ecology, and the role in malaria transmission of the species involved. This is the first study to associate spatial vector distributions with intensity of malaria transmission and parasite prevalence. The aim of this entomological study is to describe the relationship between *Anopheles* species distribution patterns and malaria incidence in the highest malaria transmission region of Colombia.

## Methods

### Study area

In order to identify and update the *Anopheles* species present in the highest malaria endemic areas of Colombia, as well to describe spatial distribution patterns and their relationship with the intensity of malaria transmission; a cross-sectional study was conducted in 70 localities, distributed in the Northwest (department of Cordoba) and South Pacific coast regions (department of Valle del Cauca and Nariño) of Colombia. These regions are two of the most important malaria endemic areas in the country. Study localities were distributed as follows: 27 in the Northwest and 43 in the South Pacific coast; names and coordinates of each locality are included in the Additional file 1a. These sites were selected based on high malaria incidence, the ease of access by land or river, and safety. A detailed description of the study areas has been previously published [15].

### Adult sampling and identification

*Anopheline* collections were performed at each locality for a week between May 2011 and November 2012. Human-landing catches were conducted indoors and outdoors in eight households at each locality. Households were selected according to convenience, taking into account: (i) presence of malaria cases, (ii) construction type, and (iii) proximity to wooded areas. Two technicians sampled each household during one night in a period of four consecutive nights, with two households sampled per night. At each household, two collectors were placed: one outdoors and another one indoors. Collections were conducted simultaneously from 18:00 to 24:00 h with collectors’ rotated hourly between indoors and outdoors settings. Samples were made during the first 50 min of every hour. All specimens collected were kept dry over silica gel, separated by site (indoors and outdoors), and date, until the mosquitoes were processed. Mosquitoes were determined using the most recent taxonomic key for *Anopheles* of Colombia [16].

### Larval sampling and identification

At each study site, all larval habitats present in a radius of 1000 m around the households selected for adult sampling were sampled for larvae, using the standard dipping method with a 400 ml ladle according to WHO procedures [17], with a sampling effort of 10 samples-dips per square metre of larval habitat. All third and fourth instars of *Anopheline* larvae were preserved in 70 % alcohol. In the laboratory, each larva was individually identified to species by morphological characters using the key for *Anopheles* of Colombia [16].

### PCR–RFLP assay

To differentiate *An. nuneztovari* s.l.*, An. rangeli,* and *An. oswaldoi* s.l., restriction profiles of internal transcribed spacer 2 (PCR–RFLP-ITS2) were used. Profiles were obtained from the amplification of this gene with the primers as proposed by Collins and Paskewitz [18]: *5,8SD* 5′-TGAACTGCAGGACACATGAA-3 and *28SR* 3′-TGCTTAAATTTAGGGGGTAGTC-5′. Posterior digestion was performed with the enzyme TaaI (HpyCH4III, Fermentas ^®^). The products were visualized using 2.5 % agarose gels. Positive controls were: DNA of *An. nuneztovari* C from department of Cordoba, confirmed by COI sequence for this study (KU925590), and DNA of *An. oswaldoi* s.l. from department of San Jose del Guaviare and *An. rangeli* from department of Meta, which had previously been confirmed by ITS2 sequence (KU945816, KU945817). The expected bands for *An. rangeli* were at 229, 104, 98 and 76 bp, for *An. oswaldoi* s.l. at 281, 233 and 18 bp, and for *An. nuneztovari* s.l. at 320 and 220 bp.

### Cytochrome c oxidase I (COI) sequencing

The DNA Barcoding region of the mitochondrial COI gene was sequenced in the sub-sample of *Anopheles* species identified by morphology. In each municipality approximately 3 % specimens of the most abundant species, and 100 % specimens of the less abundant species or species included in a complexes were selected for sequencing. The COI gene sequences were generated following the standardized methodology used for the Mosquito Barcoding Initiative of the Walter Reed Biosystematics Unit and the Natural History Museum in London to determine the degree of genetic variability and to seek evidence of species complex. DNA was extracted from the specimens following the tissue DNA extraction protocol provided by the QIAgen DNeasy blood and tissue kit (QIAGEN, Crawley, UK). All buffers were supplied in the kit. The mitochondrial gene cytochrome *c* oxidase subunit I (COI) was amplified using the primers LCO1490 (GGTCAACAAATCATAAAGATATTGG) and HCO2198 (TAAACTTCAGGGTGACCAAAAAATCA) [19] following the methodology given by Ruiz et al. [20]. PCR products were electrophoresed in 1 % Tris–acetate-EDTA buffer (TAE) and agarose gels, which were stained with ethidium bromide and cleaned using a QIAgen PCR purification kit prior to direct sequencing. PCR products were sequenced with both forward and reverse primers using an ABI-BigDye Terminator (or the Sanger dideoxy method) by the Sequencing and Analysis Service from the Molecular Genetics Institute—SSiGMol of the Universidad Nacional de Colombia in Bogota. The forward and reverse chromatograms were manually corrected using the electropherogram viewer Chromas Lite© (version 2.1.1, Technelysium Pty Ltd., Brisbane, Australia). The sequences were trimmed to 610 bp and multiple alignment was performed using Clustal W in the molecular evolutionary genetics analysis (MEGA) software version 6.0 with default parameters (open gap penalty = 15, extend gap penalty = 6.66). The resulting matrix was manually corrected.

### Natural infectivity with *Plasmodium* species

To detect natural infections with *Plasmodium* species, the head and thorax of each adult mosquito collected were used, as proposed by Foley et al. [21]. The samples were then processed following the standard protocol using the Enzyme-Linked Immunosorbent Assay (ELISA) kit distributed by the Centers for Disease Control and Prevention (CDC; Atlanta, United States), which detects the circumsporozoite protein (CS) of *Plasmodium falciparum*, *Plasmodium vivax* VK210 and *P. vivax* VK247. The cut-off used was two times the average of the negative control [22]. Positive individuals were re-tested using ELISA for accuracy. To confirm the presence of *Plasmodium* species, nested PCR analysis was performed following the methodology proposed by Snounou et al. [23]. Because the thoracic material had been used for ELISA and was no longer available, DNA was extracted from abdomens of individual mosquitoes. Briefly, two PCR reactions were performed. In the first, a region that is common to the genus *Plasmodium* was amplified with the primers 5’rPLU6 5′-TTA AAA TTG TTG CAG TTA AAA CG-3′ and rPLU5 5′-CCT GTT GTT GCC TTA AAC TTC-3′. From the amplified sample, the second PCR was performed with primers specific for *P. falciparum* species with the primers rFAL1 5′-TTA AAC TGG TTT GGG AAA ACC AAA TAT ATT-3′/rFAL 2 5-′ACA CAA TGA ACT CAA TCA TGA CTA CCC GTC-3′, and/or *P. vivax* with primers rVIV1 5′-CGC TTC TAG CTT AAT CCA CAT AAC TGA TAC-3′/rVIV2. 5′-ACT TCC AAG CCG AAG CAA AGA AAG TCC TTA-3′.

### Spatial distribution of *Anopheles* species

To describe the spatial distribution patterns, precise geographical coordinates of each immature and adult capture site were recorded using a global positioning system (Garmin GPSMAP^®^60CSx) and the distribution and proportion of *Anopheles* species collected in each locality were visualized using the software ArcGis 9.0. Later, spatial distributions of *Anopheles* species were compared with the number of malaria cases of *P. falciparum* and *P. vivax* and the annual parasite index (API) registered during 2011 and 2012 in the National System for Public Health Surveillance of Colombia- SIVIGILA [3] for the municipalities included in this study. In the South Pacific coast, the municipality of Buenaventura was divided in two areas: (1) Cali-Buenaventura road that included localities located over this road, and (2) Pacific plain localities.

### Data analysis

Natural infection rate with *Plasmodium* parasites was calculated as the percentage of mosquitoes infected. To establish the identity percent of the COI sequences obtained in this study, these were compared with available sequences in GenBank [24] and Boldsystems databases [25].

### Sequencing analysis

The COI sequences generated for specimens collected in the study sites and identified by morphological characters were aligned with the COI sequences of *Anopheles* species previously downloaded from GenBank database. All available sequences sharing the same region were included (Additional file 2); however, for the phylogram construction, some sequences were omitted. *Chagasia nr. fajardi* (GenBank: KF671013) was used as the outgroup in these analyses.

The neighbour joining (NJ) and maximum likelihood (ML) algorithms were implemented in MEGA version 6.0 program. The Tamura 3-parameter plus gamma distribution plus invariable site (T92+G+I) [26] with invariable sites (*I* = 0.54) and Gamma distribution shape parameter (*G* = 0.53) was selected as the best evolutionary distance model by MEGA 6 in model selection option [26]. Bootstrap support values (BSV) were generated by 1000 replicates. Bayesian inference (BI) was performed using MrBayes version 3.1.2. [27, 28] using default priors. Two independent analyses were run simultaneously, each with four chains and a temperature parameter value of 0.2 (the default in MrBayes). Parameters and topologies were sampled every 1000 generations. Runs were allowed to continue to 10,000,000 generations until the variance was stabilized below 0.01. Burn-in consisted of the first 25 % of generations (25,000 trees). A consensus phylogram was condensed to eliminate branch points with less than 50 % of probability.

## Results

### Anopheles species composition

**Fig. 1:**
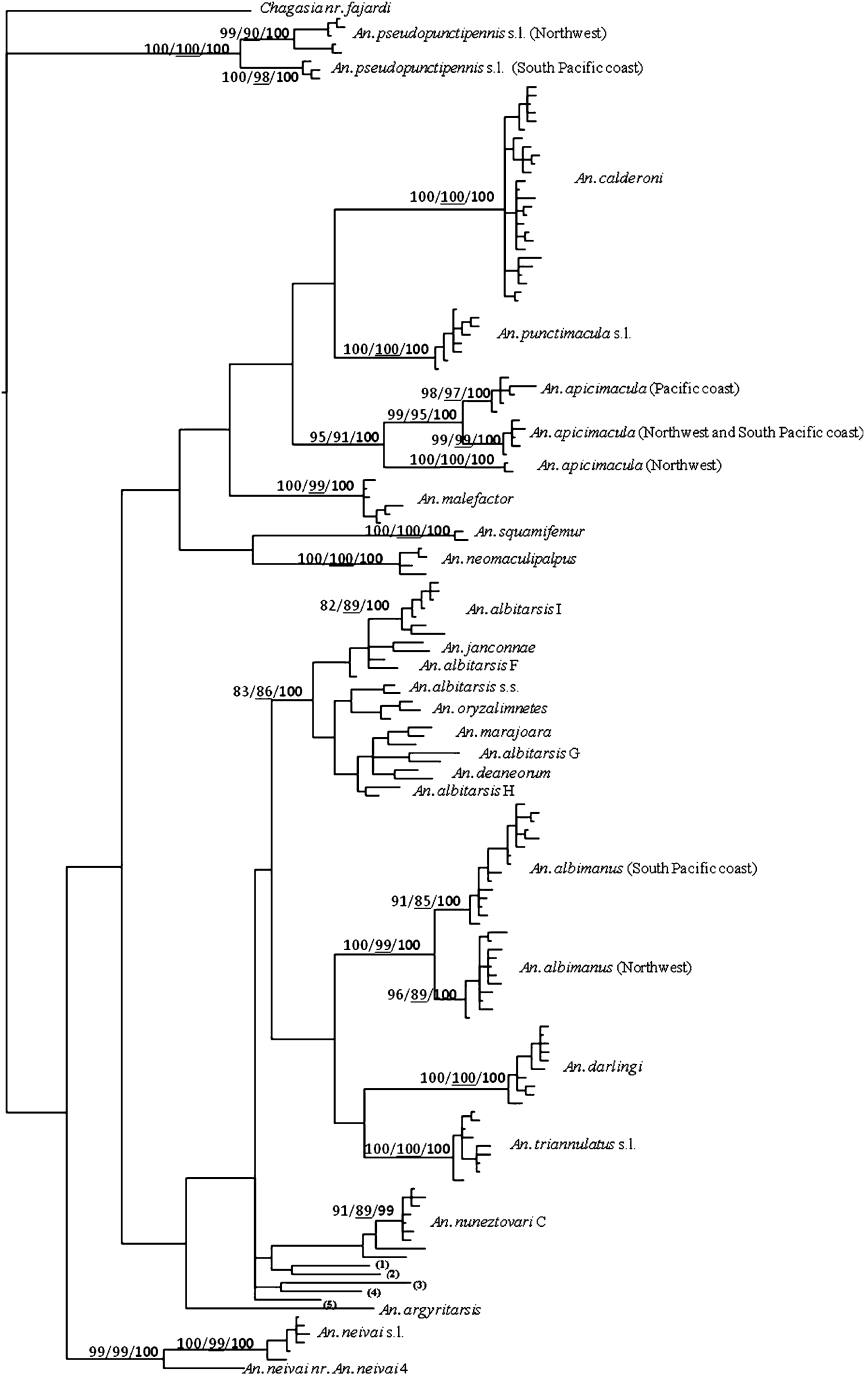
BI topology based on barcode COI sequences of *Anopheles* species identified in Northwest and South Pacific coast regions of Colombia (*First* *number* in each node indicates NJ bootstrap values (in percentages). *Second* *underline* *number* indicates ML bootstrap values. *Third* *number* in *bold* indicates Bayesian posterior probability. Outgroup taxa includes *Chagasia nr. fajardi* (GenBank KF671013). (1) *Anopheles* *nr*. *konderi,* (2) *An. oswaldoi* A, (3) *An. rangeli*, (4) *An. oswaldoi* B, (5) *An. rangeli*)

In total, 1736 larvae and 12,052 adult mosquitoes were identified in the 70 localities studied. From this entomological material, 13 species were identified using morphological characteristics and 17 mitochondrial lineages were identified using the sequence COI analysis (Tables 1, 2; Fig. 1). The DNA Barcoding region of the mitochondrial COI gene was obtained for 173 specimens: 90 from the Northwest and, 83 from the South Pacific coast region. The data of specimens used to generate the COI sequence are presented in the Additional file 1b. All sequences were deposited in the GenBank database under the following accession numbers: KU892018-KU892060, KU900755-KU900848, KU925580-KU925615).

**Table 1:** *Anopheles* species collected by human landing catches in the 70 localities of the cross-sectional study in Northwest and South Pacific regions of Colombia

Species (lineages)	Northwest region				South Pacific coast region									Total
	Cordoba				Nariño								Valle	
	Montelibano	Puerto Libertador	Tierralta	Valencia	Barbacoas	El Charco	Magui Payan	Mosquera	Olaya Herrera	Roberto Payan	Salahonda	Tumaco	Buenaventura	
*An. albimanus*	8	34	221	116	–	–	–	–	–	–	–	–	–	379
*An. albimanus* B^a^	–	–	–	–	3	175	–	3799	22	2	76	452	330	4859
*An. albitarsis* l^a^	–	–	4	1	–	–	–	–	–	–	–	–	–	5
*An. apicimacula* (NW-SPC, SPC)	–	–	1	1	–	2	–	–	–	–	–	9	2	15
*An. argyritarsis*	–	1	–	–	–	–	–	–	–	–	–	–	–	1
*An. calderoni*	–	–	–	–	4	13	–		337	58	1	46	–	459
*An. darlingi*	6	20	189	7	–	–	–	–	–	–	–	–	–	222
*An. neivai* s.l.	–	–	–	–	–	1	–	1	–	–	–	18	1.233	1253
*An. neivai nr. An. neivai* 4^a^	–	–	–	–	–	–	–	–	–	–	–	–	2	2
*An. neomaculipalpus*	–	–	–	1	–	–	–	–	–	–	–	–	–	1
*An. nuneztovari* C^a^	1026	789	1647	744	–	–	–	–	–	–	–	–	501	4707
*An. pseudopunctipennis* s.l. (NW, SPC)	–	–	12	7	–	–	–	–	–	–	–	–	22	41
*An. punctimacula* s.l.	6	–	7	2	–	–	–	–	–	–	–	–	–	15
*An. squamifemur*	–	–	–	–	–	–	–	–	–	–	–	–	2	2
*An. triannulatus* s.l.	21	29	24	17	–	–	–	–	–	–	–	–	–	91
Total	1067	873	2105	896	7	191	0	3800	359	60	77	525	2092	12,052

^a^Lineages supported in Culicidae database of Boldsystems (http://www.boldsystems.org) and GenBank databases

*NW* Northwest of Colombia, *SPC* South Pacific coast region of Colombia

**Table 2:** *Anopheles* species collected in the inspected breeding sites of the 70 localities in the cross-sectional study in Northwest and South Pacific regions of Colombia

Species (lineages)	Northwest region				South Pacific coast region									Total
	Cordoba				Nariño								Valle	
	Montelibano	Puerto Libertador	Tierralta	Valencia	Barbacoas	El Charco	Magui Payan	Mosquera	Olaya Herrera	Roberto Payan	Salahonda	Tumaco	Buenaventura	
*An. albimanus*	–	–	30	22	–	–	–	–	–	–	–	–	–	52
*An. albimanus* B^a^	–	–	–	–	–	4	–	–	4	–	8	268	1	285
*An. nuneztovari* C^a^	22	102	55	35	–	–	–	–	–	–	–	–	820	1034
*An. darlingi*	–	–	6	1	–	–	–	–	–	–	–	–		7
*An. triannulatus* s.l.	7	109	51	43	–	–	–	–	–	–	–	–		210
*An. pseudopunctipennis* s.l. (NW, SPC)	–	–	2	–	–	–	–	–	–	–	–	–	50	52
*An. calderoni*	–	–	–	–	–	2	–	–	6	–	–	40		48
*An. neomaculipalpus*	–	2	–	5	–	–	–	–	–	1	–	–	40	48
Total	29	213	144	106	0	6	0	0	10	1	8	308	911	1736

^a^Lineages supported in Culicidae database of Boldsystems (http://www.boldsystems.org) and GenBank databases

*NW* Northwest of Colombia, *SPC* South Pacific coast region of Colombia

The identity percent with sequences deposited in GenBank and Boldsystems databases was used as an initial approach for *Anopheles* species confirmation. The results are shown in the Additional file 1b. Interestingly, these results indicate that the sequences of species identified using morphological characters as *An. albimanus* from the Northwest showed a high percentage of identity (99 %) with sequences of *An. albimanus* from the Northern region of Colombia (department of Guajira); in contrast, the sequences from the South Pacific coast presented a lower identity percentage (96–97 %) with the same sequences deposited in GenBank. When the comparison in Boldsystems was conducted, all sequences of *An. albimanus* from the Northwest showed the highest percentage of identity (99.1–99.7 %) with *An. albimanus* while all sequences from the South Pacific coast showed the highest percentages of identity (99.7–100 %) with *An. albimanus* B in Boldsystems database. However, information regarding the origin of the COI sequences of *An. albimanus* B are not presented in the Boldsystem database which prevent any further analysis and comparison with the sequences from this study.

The specimens determined as *An. albitarsis* s.l. using morphological characters matched with sequences identified as “near janconnae” from the Northwest of Colombia by GenBank (98–99 % of identity) and *An. albitarsis* I from the same regions included in Boldsystems database (99.5–100 % of identity), a species also confirmed for Colombia.

With respect to *An. neivai* s.l., the COI sequences are not reported in GenBank database. The sequences from South Pacific coast (14/16) showed the highest percentage of identity with *An. neivai* in the Boldsystem records (99.7–100 %); nevertheless, in two sequences of specimens collected in the Cali-Buenaventura road, the highest percentage of identity was 94.9 % with *An. neivai* 4. It is possible that these sequences indicate the presence of another species of subgenus *Kerteszia* in this region. However, the sequences of *An. neivai* and *An. neivai* 4 are not associated with any paper to clarify the taxonomic status of these species.

*Anopheles nuneztovari* s.l. was initially confirmed using PCR–RFLP-ITS2. Of 380 specimens were included in this assay, 34 of these had been misidentified by morphological characters as *An. oswaldoi* s.l. and five as *An. rangeli*; the remaining were identified as *An. nuneztovari* s.l. The COI sequences generated for *An. nuneztovari* s.l. allowed confirmation that all specimens collected in the Northwest and the South Pacific coast regions matched with *An. nuneztovari* C (99.7–100 % of identity) registered previously by Ruiz et al. [20] in the Northeastern Colombia.

In the case of *Anopheles apicimacula* and *Anopheles squamifemur,* it was not possible to confirm their morphological determination using COI sequences. The COI sequence of *An. squamifemur* is present in neither GenBank nor Boldsystems databases, when “*Species Level Barcode Records*” option was used. However, for *An. apicimacula* and *An. squamifemur* in Boldsystems database, using “*All barcode records on BOLD*” option, 95.8–100 and 92.2–92.5 % identity percent were found respectively, but without further details of sequences origin. *Anopheles argyritarsis, An. calderoni, An. darlingi, An. neomaculipalpus, An. pseudopunctipennis* s.l., and *An. triannulatus* s.l. matched already published COI sequences in GenBank and Boldsystems databases.

The data set to sequencing analysis included 1211 COI sequences, of which 436 were used for this analysis because they shared one 610 bp barcode region fragment of which none were identical between them (see Additional file 2). Finally, 160 sequences were used to perform the COI sequences analysis presented in this paper. All sequences were checked for insertions or deletions, finding none. The sequences were translated into amino acids to identify the proper reading frame and to ensure that there were no stop codons, which would indicate the mitochondrial origin of the DNA. The final alignment of the COI gene (partial sequence) had a length of 610 bp corresponding to positions 1463 through 2071 of the *An. albitarsis* mitochondrion complete genome (GenBank: NC020662).

The ML, NJ, and BI analysis shows very similar topologies (Fig. 1), and indicates the presence of two distinctive lineages at the intraspecific level for *An. albimanus*, *An. pseudopunctipennis* s.l.*, An. neivai* s.l., and *An. apicimacula.* The two lineages of *An. albimanus* have different geographical distribution. One is found in the Northwest region, and the second is registered in the South Pacific coast, it is called *An. albimanus* B in Boldsystems database. In all phylograms, these lineages were highly supported (BVS >99). Similarly, for *An. pseudopunctipennis* s.l., the first lineage included mosquitoes collected in a Northwest region, and the second linage included COI sequences of mosquitoes collected in the South Pacific coast. These two lineages had a BVS = 100 in all analysis. The analysis of COI sequences generated for *An. neivai* s.l., collected in the South Pacific coast, shows two lineages. The first lineage, included sequences of specimens from the Cali-Buenaventura road (San Cipriano and Kilometro 24–27 localities) in the department of Valle del Cauca, matched with *An. neivai* 4 in the Boldsystems databases. The second lineage, included the remaining sequences from the South Pacific coast, identified as *An. neivai*. These lineages were supported with BVS >99 in all analysis. In the case of *An. apicimacula*, a first lineage included species collected exclusively in the South Pacific coast and the second lineage included specimens from Northwest and South Pacific coast regions; the BVS was >95 in all analysis (Fig. 1).

For *An. nuneztovari* s.l., all COI sequences generated in this study are grouped in PM, NJ, and BI analysis, with the sequences denominated *An. nuneztovari* C and reported in Northeastern of Colombia by Ruiz et al. [20]. Concerning the specimens identified by morphological characteristics as *An. albitarsis* s.l., the ML, NJ, and BI analysis, which included COI sequences of these mosquitoes and sequences for nine species reported in the Albitarsis group [6], showed that all sequences generated in the Northwest region were confirmed as *An. albitarsis* I (Fig. 1).

### Natural infection of *Anopheles* with *Plasmodium* spp. species

Four of 12,027 mosquitoes (0.03 %) were infected with *Plasmodium* spp. (Table 3). All mosquitoes found positive for CS protein were COI sequenced to confirm the *Anopheles* species. The *Anopheles* species infected with *Plasmodium* species were: *An. albimanus* B (n = 1) from the South Pacific coast infected with *P. falciparum,* and *An. nuneztovari* C (n = 3) with two specimens from the Northwest region infected with *P. falciparum* and one specimen from the South Pacific coast infected with *P. vivax* VK247. Every specimen positive by ELISA was also positive by PCR (Table 3).

**Table 3:** Number of mosquitoes processed to identify natural infection with *Plasmodium* spp. using ELISA and infection rate, obtained in 70 localities of the cross-sectional study in Northwest and South Pacific regions of Colombia

Species (lineages)	Northwest region				South Pacific coast region									# Tested	Positives ELISA	*Plasmodium* species confirmed by PCR and infection rate
	Cordoba				Nariño								Valle			
	Montelibano	Puerto libertador	Tierralta	Valencia	Barbacoas	El Charco	Magui Payan	Mosquera	Olaya Herrera	Roberto Payan	Salahonda	Tumaco	Buenaventura			
*An. albimanus*	8	34	221	116	–	–	–	–	–	–	–	–	–	379	–	
*An. albimanus* B^a^	–	–	–	–	3	174		3797	22	2	76	451	329	4854	1	*P. falciparum* (a)
*An. albitarsis* l^a^	–	–	4	1	–	–	–	–	–	–	–	–	–	5	–	
*An. apicimacula* (NW-SPC, SPC)	–	–	–	–	–	2	–	–	–	–	–	8	1	11	–	
*An. argyritarsis*	–	1	–	–	–	–	–	–	–	–	–	–	–	1	–	
*An. calderoni*	–	–	–	–	4	13	–	–	337	58	1	46	–	459	–	
*An. darlingi*	6	20	189	7	–	–	–	–	–	–	–	–	–	222	–	
*An. neivai* s.l.	–	–	–	–	–	1	–	–	–	–	–	18	1232	1251	–	
*An. neivai nr. An. neivai* 4^a^	–	–	–	–	–	–	–	–	–	–	–	–	2	2	–	
*An. nuneztovari* C^a^	1025	786	1646	742	–	–	–	–	–	–	–	–	500	4699	3	2 *P. falciparum* (b); 1 *P.vivax* VK247 (c)
*An. pseudopunctipennis* s.l. (NW, SPC)	–	–	12	7	–	–	–	–	–	–	–	–	22	41	–	
*An. punctimacula* s.l.	4		7	2	–	–	–	–	–	–	–	–	–	13	–	
*An. triannulatus* s.l.	21	28	24	17	–	–	–	–	–	–	–	–	–	90	–	
Total	1064	869	2103	892	7	190	0	3797	359	60	77	523	2086	12,027	4	0.03

^a^Lineages supported in Culicidae database of Boldsystems (http://www.boldsystems.org) and GenBank databases

*NW* Northwest of Colombia,* SPC* South Pacific coast region of Colombia. (a) El Charco, Nariño, (b) Tierralta, Cordoba, (c) Buenaventura, Valle del Cauca

### Spatial distribution of *Anopheles* species in the states of study and its relation to malaria incidence

**Fig. 2:**
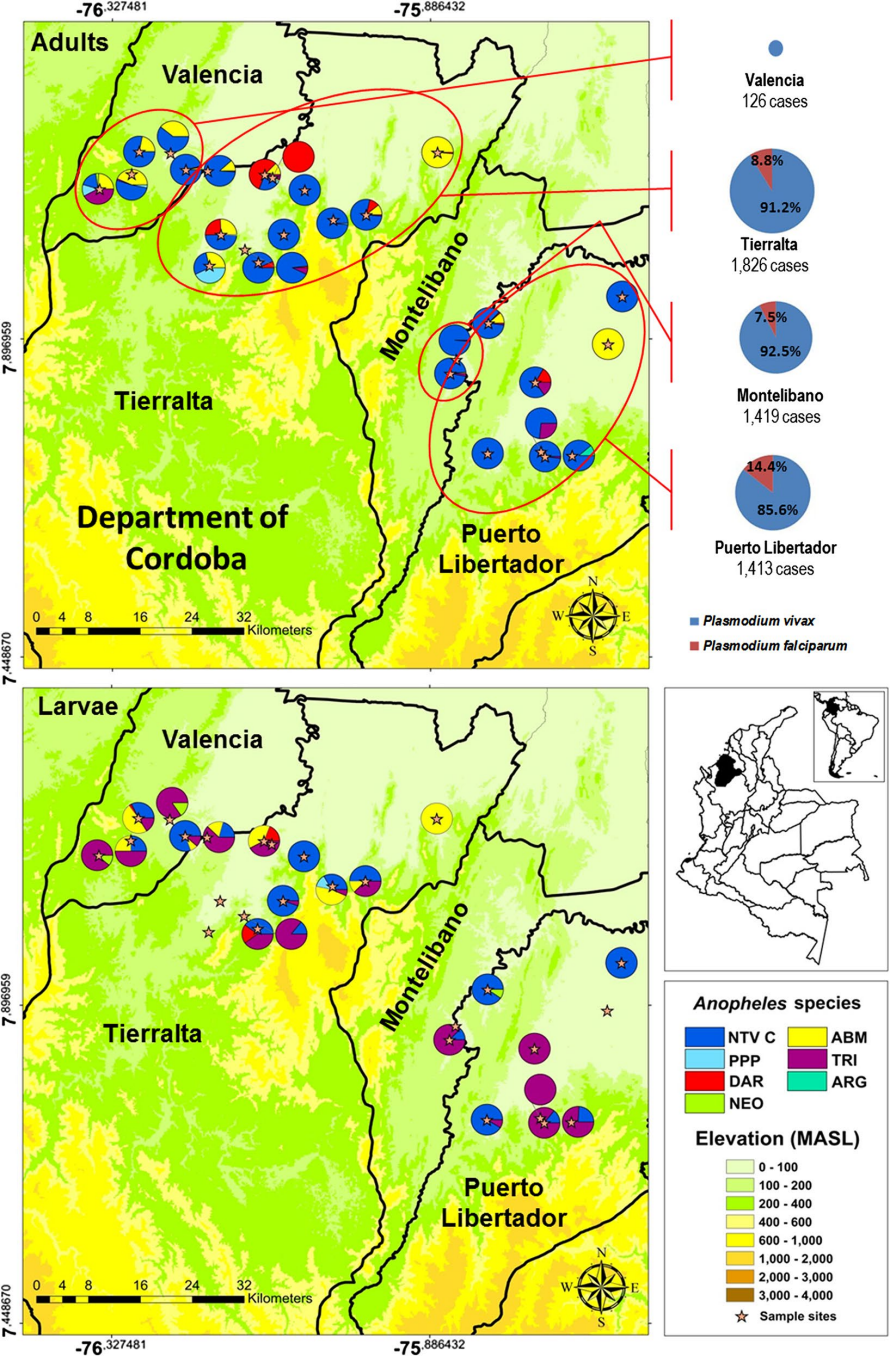
Distribution of *Anopheles* species found in the Northwest region of Colombia and malaria cases reported. (*NTV* *An. nuneztovari* C; *ABM* *An. albimanus*; *DAR* *An. darlingi*; *PPP* *An. pseudopunctipennis* s.l.; *TRI* *An. triannulatus* s.l.; *ARG* *An. argyritarsis*)

Regarding the number of malaria cases reported and malaria incidence in the year of sampling, in the Northwest region, particularly in the department of Cordoba (with four municipalities included in this study), the municipality of Valencia reported the lowest number of cases (126), with an annual parasite incidence (API) of 5 cases/1000 inhabitants; while the municipalities of Tierralta, Montelibano, and Puerto Libertador reported a similar number of cases each: 1826 (API: 35 cases/1000 inhabitants), 1419 (API: 86 cases/1000 inhabitants) and 1, 413 (API: 55 cases/1000 inhabitants), respectively. The proportion between *P. vivax* and *P. falciparum* infections was similar in the municipalities of study except in Valencia where all infections were only by *P. vivax* (Fig. 2). In the Northwest region, the most widely distributed species for both adults and larvae was *An. nuneztovari* C. Other malaria vectors found were *An. albimanus* and *An. darlingi* (Fig. 2).

**Fig. 3:**
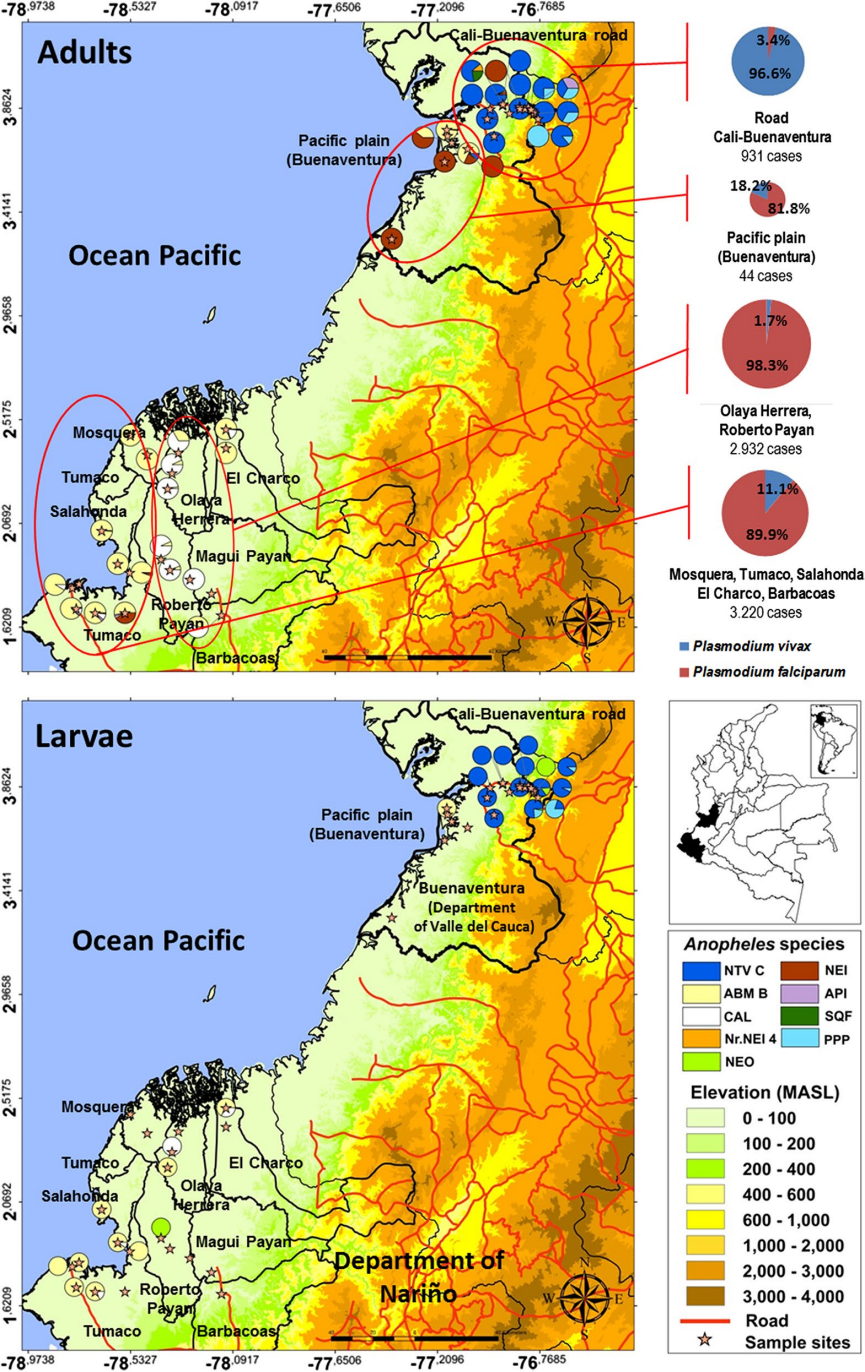
Distribution of *Anopheles* species found in the South Pacific coast region of Colombia and malaria cases reported (*NTV* *An. nuneztovari* C; *NEI* *An. neivai* s.l.; Nr. NEI 4: *An. neivai nr*. *An. neivai* 4; *ABM* B *An. albimanus* B; *API* *An. apicimacula;* *NEO* *An. neomaculipalpus*; *PPP An. pseudopunctipennis* s.l.; *SQF* *An. squamifemur*)

The South Pacific coast is comprised of two areas: (1) the inland area along the Cali-Buenaventura road that includes the foothills of The Andes mountains and, (2) the Pacific plain area that involve the southern portion of Buenaventura and ten municipalities of the department of Nariño. In the first, *P. vivax* was more prevalent and in the second, *P. falciparum* was the more prevalent species. In the municipality of Buenaventura, located in the north of this region, an API of 30 cases/1000 inhabitants was registered in the years of mosquito collection. In this area, *An. nuneztovari* C was the *Anopheles* species most widely distributed in adult stage, and *An. pseudopunctipennis* s.l. (Pacific coast lineage) was found in the villages located at the foothills (Fig. 3). In the coastal study sites of Pacific plain area, the predominant species were *An. albimanus* B (Pacific coast lineage) and *An. neivai* (*An. neivai* lineage). In inland in the department of Nariño, *An. calderoni* was the most important species. A different incidence was observed in the area where *An. albimanus* B predominates, in which 3220 cases and an API of 27 cases/1000 inhabitants were reported, as compared with the area where *An. calderoni* predominates, in which 2932 cases and an API of 73 cases/1000 inhabitants were reported (Fig. 3).

## Discussion

During this study, using PCR–RFLP-ITS 2 and morphological characters, 13 formally named *Anopheles* species were confirmed in the endemic malaria region of Colombia, however, the NJ, ML, and BI analysis using COI barcode sequences genes revealed the presence of four new mitochondrial lineages. One new lineage of *An. albimanus,* called *An. albimanus* B in the Boldsystem databases, was found in the South Pacific coast. One new lineage of *An. pseudopunctipennis* s.l., was found in the Northwest and the South Pacific coast. One new lineage for *An. neivai* (*An. neivainr. An. neivai* 4) was found in South Pacific coast and one new lineage for *An. apicimacula* that include sequences from Northwest and South Pacific coast regions.

Regarding the association of *Anopheles* species with malaria distribution, the department of Cordoba, located in the Northwest region of Colombia, contributes with 18 % of malaria cases reported annually in the country [2]. This region was the study area with the highest number of *Anopheles* species, ten out of the 13 species identified. An important aspect of the current study is that in this area three primary malaria vectors were registered: *An. nuneztovari* s.l. (=*An. nuneztovari* C)*, An. darlingi* and *An. albimanus* (northwest lineage). This may partly explain the local high malaria burden. Both *An. nuneztovari* s.l. and *An. darlingi* are anthropophilic species with an important biting activity in evening early hours [29–38]. During this period, people are still active and unprotected by LLINs, the main measure used for the control of malaria vectors in this region. Interestingly, the results show significant differences in relation to the predominant species in adult stage and immature stage. Predominant species in adults was *An. nuneztovari* C (85 % of collections), whereas *An. nuneztovari* C and *An. triannulatus* s.l. were collected in similar proportions in immature stages. Differences in *Anopheles* species composition in adult and immature stages may be explained by the presence of cows that provides blood sources for the zoophilic species like *An. triannulatus* s.l. [39–41].

With respect to another *Anopheles* species registered in the Northwest region, in this study, the presence of *An. albitaris* I (n = 5) was confirmed. This species had been reported in the same region by Gutierrez et al. [42] as *An. albitarsis* near janconae; however, afterwards it was confirmed by Ruiz et al. [6] as *An. albitaris* I. According to the results of this study, the distribution of *An. albitaris* I was extended to others localities in the Northwest region. The malaria vector status of *An. albitaris* I is unknown in South America [6]. Additionally, morphological characters and COI sequence analysis confirmed the presence of other *Anopheles* species in low densities: *An. pseudopunctipennis* s.l. (northwest lineage) (n = 19), *An. punctimacula* s.l. (n = 16), *An. apicimacula* (northwest lineage) (n = 1), *An. argyritarsis* (n = 1), and, *An. neomaculipalpus* (n = 1).

In relation to the role that *Anopheles* species play in malaria transmission in the Northwest region, three primary vectors were registered: *An. nuneztovari* C*, An. darlingi* and *An. albimanus*. In this study, two specimens of *An. nuneztovari* C were found infected with *P. falciparum*; however, previous studies conducted in the same region reported *An. nuneztovari* s.l. as infected with *P. vivax* VK247. The taxonomic determination of species within this species complex was not clarified in these studies [34, 43]. It is likely that the species corresponded to *An. nuneztovari* C from the results found in the present study. Other species reported as positive for *Plasmodium* infection were *An.darlingi* infected with *P. vivax* VK247 [34, 43, 44], and *An. triannulatus* s.l. infected with *P. vivax* VK247 [43]; however, the role of the latter species in the Northwest region is not yet clear. Other *Anopheles* species registered in Northwest region, *An. pseudopunctipennis* s.l., *An. punctimacula* s.l., and, *An. neomaculipalpus* are considered malaria vector in America [45–47], however, in this region their importance in the sustaining of malaria transmission is unknown.

During the past ten years, in Buenaventura (department of Valle del Cauca), API fluctuated between nine and 167 cases/1000 inhabitants and in ten municipalities of department of Nariño, with the highest malaria transmission, this index ranged between 22 and 100 cases/1000 inhabitants, with a prevalence of *P. vivax* inland, along the Cali-Buenaventura road, and *P. falciparum* in the Pacific plain in the South Pacific region. In this region, seven *Anopheles* species were identified. The most abundant *Anopheles* species in the area along the Cali-Buenaventura road were *An. nuneztovari* C and *An. pseudopunctipennis* s.l. (Pacific coast lineage). *Anopheles neivai* s.l. was identified with evidence of two lineages (*An. neivai* and *An. neivainr*. *An. neivai* 4). In the Pacific plain area, *An. albimanus* B was the most important species along the coast; while inland *An. calderoni*, *An. neivai,* and *An. apicimacula* (Northwest-Pacific coast and only Pacific coast lineages) were confirmed. Different composition in adults and larval stages of *Anopheles* species were observed in the South Pacific coast. As evidenced in the maps (Fig. 3), in coastal localities *An. albimanus* B and *An. neivai* were registered in adult state, whereas inland *An. nuneztovari* C, *An. pseudopunctipennis* and *An. calderoni* were registered. *Anopheles neivai* were collected in adult but not in immature states in the coastal towns. This can be explained by the difficulty of sampling in the water that is accumulated in the axils of bromeliads leaves, *An. neivai* s.l. larval habitat [48–51]. Sampling of bromeliads could only be carried out in three localities where the species was present in the adult stage. In general, these Colombian Pacific coastal localities are surrounded by mangroves, which host several species of bromeliads.

In the study sites of the South Pacific coast, two specimens were found infected with *Plasmodium* species: *An. albimanus* B from the Pacific plain region was infected with *P. falciparum;* and *An. nuneztovari* C from the Cali-Buenaventura road was infected with *P. vivax* VK247. In various studies carried out in recent years in the same region, *An. albimanus* was reported infected with *P. falciparum* [52] and with *P. vivax* VK210 [52, 53]. Additionally, in this region another two *Anopheles* species have been reported infected with *Plasmodium:Anopheles neivai* infected with *P. vivax* VK210, *P. vivax* VK 247 and *P. falciparum* [52, 53], and *An. calderoni* infected with *P. vivax* VK210 and *P. falciparum* [14, 44]. The previous results provides evidence to confirm the status as malaria vectors of: *An. nuneztovari* C, *An. albimanus* B, An*. neivai,* and *An. calderoni* in the Southwest of Colombia, due to their presence and wide distribution in a region with active malaria transmission, anthropophilic behaviour, and detection of natural infection with *Plasmodium* species [14, 44, 53–55].

Although this study included data about *Anopheles* species composition at locality level, the relationship between the presence of *Anopheles* species and the specific locality burden of malaria was difficult to determine, due mainly to difficulties in the reporting system. Although, in Colombia it is mandatory to report malaria cases by locality of origin, it is common to find the locality origin of the infection mixed up with the locality where the patient lives, or with the locality in which the diagnosis was made. Also, patients could refuse to give information about the locality in which they probably got the infection. These situations make the analysis by localities difficult. For this reason, the relation between *Anopheles* species composition and malaria prevalence is presented at municipality level.

*Anopheles* species with two lineages identified in this study were: *Anopheles albimanus*, *An. pseudopunctipennis* s.l., *An. neivai* s.l., and *An. apicimacula.* Some considerations about taxonomic status are put forward here: *Anopheles albimanus* shows two lineages: Northwest, *An. albimanus* and Pacific coast, *An. albimanus* B, each lineage with different geographical distribution. The differences between *An. albimanus* populations of the Caribbean regions (North of Colombia) and the Pacific coast had previously been reported by Narang et al. [56] using allozyme variability and chromosomal analysis to characterize *An. albimanus* populations collected in 11 localities from Colombia (north and Pacific coast), and were confirmed by Gutierrez et al. [57], who defined two distinctive groups corresponding to haplotypes from the Caribbean and Pacific coast regions by analysis of COI and microsatellite. However, the reported data of hybridization and backcrosses that included North and South Pacific coast populations of *An. albimanus,* showed that hybrid females and males were fertile and had normal ovaries and testes, indicating the absence of cryptic speciation for this species in Colombia [56]. It is necessary to carry out studies in vector competence to identify differences in infection susceptibility by strains of *Plasmodium*, because both lineages are present in regions with different malaria prevalence.

Similarly, evidence to support two lineages of *An. pseudopunctipennis* s.l. with different geographical distribution was found: one linage conformed by specimens collected in the Northwest region and the other by specimens collected from the Southwest in the Pacific region. Several studies have attempted to evaluate the taxonomic status of *An. pseudopunctipennis* s.l. Estrada-Franco et al. [58] used isoenzyme electrophoresis and rDNA restriction fragment length polymorphisms (RFLPs) and Coetzee et al. [59] used cross-mating experiments, suggested that this species is actually a complex of three species: *An. pseudopunctipennis* A, represented by specimens from central Mexico; *An. pseudopunctipennis* B, represented by specimens from the Andes of Peru and Bolivia [58]; and *An. pseudopunctipennis* C, represented by specimens collected on the island of Grenada in the Lesser Antilles [59]. However, based on the evidence of isozyme analyses of 42 populations of this species collected in ten countries in North, Central, and South America, Manguin et al. [60] suggested that *An. pseudopunctipennis* s.l. is a single species with three geographical populations represented in: (1). North America (United States and Mexico) and Guatemala; (2). Belize and South America (Colombia, Ecuador, Peru, Chile, and Argentina); and (3). Grenada Island. Additionally, Manguin et al. [60] suggested that the first and second geographic population converge in Southern Mexico and Northern Central America on the border between Belize and Guatemala. However, it should be noted that in the study by Manguin et al. [60], the samples included for analysis from Colombia came from the South of the country with no representation of specimens of this species from the North, which would probably be more like those found in Central America: a hypothesis that should be tested.

*Anopheles neivai* s.l. was another species that showed two lineages; however, in contrast to *An. albimanus* and *An. pseudopunctipennis* s.l., these lineages were found in the same region. The limitation of this finding is that all specimens identified by morphological characters as *An. neivai* s.l., in localities where the second lineage (*An. neivai nr*. *An. neivai* 4) is present, were not sequenced and the lineage *An. neivai* were confirmed in the same area, leaving untested the sympatry of the two lineages in the South Pacific coast region.

Regarding *An. apicimacula,* the findings partially support the results reported by Gomez et al. [61] who inferred the existence of two lineages, Atlantic coast and Pacific coast. The results of the present study show two lineages with a BVS >95, the first included the COI sequence of specimens collected exclusively in Pacific coast, and the second included the sequence generated for specimens collected in the Northwest and the South Pacific coast. In the same sequence analysis, a third lineage was observed with a BVS = 100, which only included sequences generated by Gomez et al. [61] using specimens collected in the Northwest region, which were grouped in the original study as *An. apicimacula* Caribbean lineage (Fig. 1). These results suggest that is necessary perform an analysis that includes more COI sequences of Northwest region to clarify because the second lineage include the Northwest and South Pacific coast sequences and confirm the different populations of *An. apicimacula* present in Colombia.

This study provides evidence about the richness of *Anopheles* species found in malaria transmission regions in Colombia and establishes that several species incriminated as malaria vectors are sympatric. This condition is an important aspect to consider designing control strategies, given the fact that species can exhibit different biting behaviours that could diminish the effectiveness of traditional control measures when applied to large scale.

## Conclusions

In the malaria endemic areas of the Northwest and South Pacific coast regions of Colombia, 13 *Anopheles* species and four new lineages were confirmed using COI sequences analysis. The DNA barcode analyses refine the taxonomic identification process, particularly for species complexes, which can lead to advancements in the understanding of the relationship between species complexes and malaria transmission. The result provides evidence about *Anopheles* species richness, and the different composition and relative abundance presented in each study regions. In the Northwest and South Pacific regions two dominant vector species were identified, *An. nuneztovari* C and *An. albimanus* B. Both were naturally infected with *Plasmodium* species. However, other species present in low abundance have also been incriminated as malaria vectors in Colombia (9, 14), such as *An. darlingi*, *An. calderoni* and *An. neivai* s.l., and they might also sustain malaria transmission in these regions. The knowledge of local vector distribution will be useful for planning targeted intervention strategies for malaria control and elimination.

## Additional files

10.1186/s12936-016-1421-4 a: Localities included in a cross sectional study in Northwest and South Pacific coast regions. b: List of *Anopheles* specimens using to generate COI sequence and identity percent with COI sequences deposited in GenBank and Boldsystems databases.
10.1186/s12936-016-1421-4 *Anopheles* species and GenBank accession numbers of COI sequences used to sequence analysis. In bold accession numbers assigned to COI sequences generated in this study by GenBank database.

## Abbreviations

API: annual parasitic index
BI: bayesian inference
BLAST: basic local alignment search tool
BOLD: barcode of life data system
bp: base pair
BVS: bootstrap support value
CDC: Centers for Disease Control and Prevention
CLAIM: Latin American Research Center in Malaria
COI: cytochrome *c* oxidase subunit I gene
CS: circumsporozoite protein
DNA: deoxyribonucleic acid
ELISA: enzyme-linked immunosorbent assay
IRS: indoor residual spraying of insecticides
ITS2: internal transcribed spacer 2
LLIN: long-lasting insecticidal nest
MEGA: molecular evolutionary genetics analysis
ML: maximum likelihood
NIH: National Institutes of Health
NJ: neighbour joining
nr: near
PCR: polymerase chain reaction
RFLP: restriction fragment length polymorphisms
RFLP-ITS2: restriction profiles of the internal transcribed spacer 2
SEM: Servicio de Erradicación de la Malaria
SIVIGILA: Sistema Nacional de Vigilancia en salud pública de Colombia
TAE: tris-acetate-EDTA buffer
WHO: World Health Organization
