# Supplementary material for: Spatial distributions of Anopheles species in relation to malaria incidence at 70 localities in the highly endemic Northwest and South Pacific coast regions of Colombia
Source: Malar J. 2016 Aug 11;15:407. doi: 10.1186/s12936-016-1421-4 (PMC4981953; doi:10.1186/s12936-016-1421-4)
Supplement: Supplementary file 2 — 10.1186/s12936-016-1421-4 Anopheles species and GenBank accession numbers of COI sequences used to sequence analysis. In bold accession numbers assigned to COI sequences generated in this study by GenBank database. [file 12936_2016_1421_MOESM2_ESM.docx]

**Additional file 2.** *Anopheles* species and GenBank accession numbers of COI sequences used to sequence analysis. In **bold** accession numbers assigned to COI sequences generated in this study by GenBank.

| ***Anopheles* species** | **GenBank accession numbers sequences of COI** |
| --- | --- |
| *An. albimanus/An. albimanus* B | KC354823, KC354824, **KU892033-KU892042**, **KU900784-KU900794**, **KU900805-KU900808**. |
| *An. albitaris* F | JQ615000, JQ615002, JQ615005-JQ615013, JQ615015-JQ615017, JQ615019- JQ615021, JQ615024, JQ615026-JQ615038, JQ615040. |
| *An. albitaris* H | JQ615148-JQ615151, JQ615153, JQ615156, JQ615157, JQ615159, JQ615163, JQ615164, JQ615166- JQ615170, JQ615173, JQ615175- JQ615177, JQ615181, JQ615187. |
| *An. albitarsis* G | JQ615045, JQ615046, JQ615049, JQ615052, JQ615057, JQ615059, JQ615062, JQ615070, JQ615075, JQ615082-JQ615084, JQ615087, JQ615091, JQ615092, JQ615096, JQ615098, JQ615099, JQ615110, JQ615114, JQ615116, JQ615131, JQ615133, JQ615134, JQ615136, JQ615141, JQ615145. |
| *An. albitarsis* I | JQ615190, JQ615191, JQ615193, JQ615196, JQ615198, **KU892045-KU892049**. |
| *An. albitarsis* s.s. | JQ615202-JQ615204, JQ615206, JQ615210-JQ615219, JQ615221-JQ615237, JQ615239-JQ615241, JQ615243, JQ615246- JQ615254. |
| *An. apicimacula* | KF698866-KF698868, KF698870-KF698872, **KU900813-KU900817**, **KU900756**. |
| *An. argyritarsis* | **KU892050**. |
| *An. calderoni* | HQ642968-HQ642970, HQ642972-HQ642974, KF698802, KF698804-KF698820, KF698822-KF698824, KF698826-KF698832, **KU892018-KU892030**. |
| *An. darlingi* | HM022406, KP193458, DQ076235, DQ076236, JF923693, JF923695, KC555065, **KU892051-KU892055**. |
| *An. deaneorum* | JQ615310-JQ615334, JQ615336-JQ615340, JQ615342-JQ615345. |
| *An. janconnae* | JQ615346, JQ615349, JQ615356-JQ615358, JQ615361, JQ615365-JQ615371, JQ615375, JQ615377-JQ615379, JQ615381, JQ615383, JQ615385, JQ615388, JQ615392. JQ615395, JQ615396, JQ615399, JQ615401, JQ615403-JQ615405, JQ615409, JQ615410, JQ615414, JQ615416, JQ615418, JQ615419, JQ615422, JQ615425, JQ615430- JQ615433, JQ615437, JQ615440. |
| *An. malefactor* | KF698825, KF698838-KF698842. |
| *An. marajoara* | JQ615458, JQ615460, JQ615464, JQ615466, JQ615471, JQ615474, JQ615476, JQ615478, JQ615481, JQ615483, JQ615494, JQ615496, JQ615501, JQ615508, JQ615442. |
| *An. neivai* s.l. | **KU900811**, **KU900820-KU900822**, **KU900826-KU900828**. |
| *An. neomaculipalpus* | KF698843- KF698854, KF698856- KF698862, **KU900755**. |
| *An. nr konderi* | KF809133. |
| *An. nuneztovari* s.l. | HQ315869-HQ315872, HQ315874, HQ315875, HQ315877, JF923710, JF923711, JF923713, **KU925580-KU925584**, **KU925604**. |
| *An. oryzalimnetes* | JQ615513, JQ615520, JQ615527, JQ615529-JQ615532, JQ615534, JQ615536, JQ615538, JQ615541, JQ615544, JQ615546-JQ615549, JQ615551, JQ615554, JQ615557, JQ615561. |
| *An. oswaldoi* A | KF809079. |
| *An. oswaldoi* B | KF809105, KF809109. |
| *An. pseudopunctipennis* s.l. | **KU900834-KU900838**, **KU900845-KU900847**. |
| *An. punctimacula* s.l. | HQ622626, KF698834-KF698837, **KU900757-KU900759**. |
| *An. rangeli* | JX205121, JF923725. |
| *An. squamifemur* | **KU900771**, **KU900772**. |
| *An. triannulatus* s.l*.* | **KU900773-KU900781**. |
